# The health impact of remarriage behavior on chronic obstructive pulmonary disease: findings from the US longitudinal survey

**DOI:** 10.1186/1471-2458-9-412

**Published:** 2009-11-14

**Authors:** Tatsuya Noda, Toshiyuki Ojima, Shinya Hayasaka, Akihito Hagihara, Ryoichi Takayanagi, Koichi Nobutomo

**Affiliations:** 1 Department of Community Health and Preventive Medicine, Hamamatsu University School of Medicine, Handayama 1-20-1, Hamamatsu city, Shizuoka, Japan; 2 Department of Health Care Administration and Management, Kyushu University Graduate School of Medicine, Maidashi 3-1-1, Fukuoka city, Fukuoka, Japan; 3 Department of Medicine and Bioregulatory Science, Kyushu University Graduate School of Medicine, Maidashi 3-1-1, Fukuoka city, Fukuoka, Japan; 4 Department of Health Services Management and Policy, Kyushu University Graduate School of Medicine, Maidashi 3-1-1, Fukuoka city, Fukuoka, Japan

## Abstract

**Background:**

Chronic obstructive pulmonary disease (COPD) is a major disease among adults, and its deterioration was reported to be associated with psychological imbalance. Meanwhile, bereavement and divorce have proven harmful to the health status of a surviving spouse. But few studies have been conducted to evaluate the remedial effect on survivors' health outcome by remarriage after bereavement. The present study thus examined the associations between remarriage and the onset of COPD.

**Methods:**

Our cohort was drawn from Health and Retirement Study participants in the United States, and consisted of 2676 subjects who were divorced or bereaved from 1992 to 2002. We then followed them for up to 11 years and assessed the incidence rate of COPD using a Cox proportional hazard model after adjusting for marital status, age, gender, education and the number of cigarettes smoked.

**Results:**

Among all subjects, 224 who remarried after bereavement or divorce tended to be younger and more male dominated. Remarriage after bereavement/divorce was associated with significantly decreased risk of COPD onset for overall subjects [hazard ratio (HR): 0.51, 95% confidence interval (95% CI): 0.28-0.94], female subjects [HR: 0.36, 95% CI: 0.13-0.98], and for those under 70 years old [HR: 0.36, 95% CI: 0.17-0.79].

**Conclusion:**

This study investigates the impact of remarriage on health outcome based on a large-scale population survey and indicates that remarriage significantly correlates with reduced risk of COPD incidence, even after adjusting smoking habit.

## Background

Losing a family member is one of the most serious life transitions for many adults. Studies on the health impact of bereavement have been conducted from the nineteenth century and still remain an important issue [1-7]. The harmful effect of bereavement on a marriage partner is almost certain from previous nationwide studies [8,9]. Although the bereavement effect is more obvious among younger than older people, more than 600,000 adults are widowed each year in the United States and the bereavement effect in later years brings about a growing impact upon aging society [10,11]. Spousal loss in later life is irreparable and unavoidable unless one divorces or dies before a partner, so it is meaningful to seek for modifiable social factors which might soften the impact of late life spousal bereavement. In this study, we focused on remarriage after bereavement as a possible rescue factor against the bereavement effect because remarriage is a joyful event in general and remarriage is possible after the onset of bereavement.

From the viewpoint of spousal loss, divorce or separation among couples also seems to be an important event in later life. In the United States, more than 1.1 million people over 50 have been divorced or separated, and it is about 6 times greater in those who have been widowed[11] Although the magnitude and direction of the impact by divorce varies with the individual, for those shocked by divorce, remarriage is a possible relief event. But few studies have been conducted on the relationship between remarriage after divorce and the alteration of health statistics, and it remains unclear whether remarriage would make the health of those divorced take a turn for the better [12,13].

Several studies have examined the effect of remarriage on health status [14-17]. While two longitudinal studies suggested that remarriage might reduce mortality after bereavement, another suggested that remarriage after divorce was not associated with health status [4,18,19]. To our knowledge, no study has been found which focuses on the association between remarriage and incidence rates of particular diseases.

In the present study, we examined the relationship between remarriage after bereavement and the onset of disease, especially chronic obstructive pulmonary disease (COPD) which is known to have a large negative impact on health-related quality of life in the elderly [20,21]. Although smoking habits are well known as a major risk factor of COPD, functional impairment in this disease occurs in correlation with anxiety and depression in the older adults [22]. It remains possible that the psychological burden in pre-COPD patients aggravates their respiratory function and acts as a trigger for COPD onset.

Most previous studies on bereavement and mortality were limited because they were based on demographic data and therefore did not take into account the subjects' health status and health-related behavior. It is likely that people with unhealthy behavior are less likely to remarry than healthy people [23]. In this study, we succeeded both in excluding those who were already COPD patients from our study population and in including subjects' health behavior (i.e. their smoking status) into our study model.

## Methods

### Study population

The Health and Retirement Study (HRS) is a nationally representative longitudinal study sponsored by the National Institute on Aging [24]. The HRS sample is selected using a multi-stage area probability sample design. The HRS consisted of two cohorts. The original HRS cohort began in 1992 noninstitutionalized adults in the 48 states of the United States who were born during 1931-1941, with oversampling of blacks and Hispanics and Florida residents. The other cohort is Asset and Health Dynamics Among the Oldest Old cohort started in 1993 with adults born in 1923 or earlier. If a subject married, a new spouse was included in the cohort. In both cohorts, a survey has been conducted every 2 or 3 years (usually biennially). As a consequence, HRS included 30888 participants from 1992 to 2004. All respondents provided oral informed consent, and internal ethics review board approval was obtained. More detailed information regarding the HRS design can be available in the special feature paper [24]. After applying for registration as a data user and obtaining permission from the Health and Retirement Study group, we conducted analyses with the use of publicly available data files.

From this HRS cohort, we selected 4100 subjects who experienced divorce or bereavement and then excluded 302 subjects who dropped out of the HRS shortly after they divorced or were bereaved, 522 subjects who did not answer all the questions required and 600 subjects who had already developed COPD at study entry. Thus, we eventually identified 2676 subjects for the analysis.

### Outcome and exposures

While subjects who would have never remarried until censoring or the end of follow-up were included in the not-remarried group, subjects who remarried in the follow-up period entered the remarried group. Subjects were censored when they died or were lost to follow-up (in both groups), or when they were widowed by or divorced from their partner (in the remarried group). As each survey of the HRS was performed every two or three years, the midpoint between the two consecutive surveys was set as the time of event occurrence.

Our primary endpoint was the incidence rate of COPD. Marital status after divorce/bereavement, age at study entry, gender, race/ethnicity (white/Caucasian, or other), education (none/basic/secondary, or high school and higher), and the number of cigarettes smoked at study entry were included in the study model as potential confounding variables. Age at study entry was treated as a continuous variable, and the number of cigarettes was classified into 3 categories (non-smoker, 1-20 cigarettes per day and over 20 cigarettes per day), while the remaining variables were modeled as binary covariates.

The HRS questionnaire asked respondents whether a doctor had ever told them that they had chronic lung disease such as chronic bronchitis or emphysema. Although precise definitions of COPD vary, COPD most commonly refers to patients with chronic bronchitis and emphysema [25] We thus determined the onset of COPD based on the questionnaire noted above. The HRS questionnaire, in addition, equated a common-law partnership with official marriage.

### Statistical analyses

Significance tests were performed for age at entry and all other variables using Student's *t *test and chi-square tests, respectively. The Cox proportional hazard model was used to calculate the hazard ratios of COPD onset. To test for parallelism, the proportional assumption was graphically verified by plotting log [-log (survivor function)] against time in groups identified by each covariate. Statistical analyses were performed using SAS version 9.1.3.

## Results

The baseline characteristics of the subjects who remarried and those who remained single (not-remarried) are shown in Table 1. These groups yielded 12954.5 person-years from 2676 individuals. At study entry, subjects were aged 61.8 years on average and were 56.3% male in the remarried group, while, in the not-remarried group, they were aged 69.9 years on average and 30.3% male. Subjects in the remarried group were significantly younger and more male dominated than those in the not-remarried group. In the remarried group, the proportions of subjects whose race/ethnicity was white/Caucasian, with higher education (*i.e*., having a high school diploma or higher) and non-smoker were 84.4%, 31.3%, and 84.3%, respectively, while, in the not-remarried group, these were 82.5%, 35.8%, and 87.4%, respectively. No significant differences between both groups were observed in the proportion of male sex, white/Caucasian, those having a higher education and smoking level. Of all 2676 subjects, 183 (6.8%) developed COPD. Compared with the two groups, the number of affected subjects was 12 (5.4%) in the remarried group and 171 (7.0%) in the not-remarried group. While 122 (6.8%) male subjects developed COPD, 61 (7.0%) female subjects did so. No significant differences were observed with either crude comparison.

**Table 1:** Baseline characteristics of subjects

	Remarried (n = 224)	Not-remarried (n = 2452)	Two-sided p value
**Age at study entry**			
mean age (± SD)	61.8 (± 9.7)	69.9 (± 11.6)	< 0.001
**Sex**			
male	126 (56.3%)	744 (30.3%)	< 0.001
female	98 (43.8%)	1708 (69.7%)	
**Race/ethnicity**			
white/Caucasian	189 (84.4%)	2023 (82.5%)	0.482
others	35 (15.6%)	429 (17.5%)	
**Education**			
none/basic/secondary	70 (31.3%)	878 (35.8%)	0.176
high school or higher	154 (68.8%)	1574 (64.2%)	
**Number of cigarettes smoked at study entry**			
none	186 (83.0%)	2142 (87.4%)	0.176
1-20 cigarettes/day	31 (13.8%)	258 (10.5%)	
> 20 cigarettes/day	7 (3.1%)	52 (2.1%)	
**COPD onset during follow-up**			
	12 (5.4%)	171 (7.0%)	0.359

Data are number unless otherwise indicated.

The Cox proportional hazards model was used to examine the effect of remarriage after divorce or bereavement on the incidence rate of COPD (Table 2). In overall analysis, remarriage after divorce or bereavement was significantly correlated with a lower incidence rate of COPD (hazard ratio: 0.51). On the contrary, white/Caucasian race, lower education level and smoking more cigarettes were significantly associated with the increased incidence of COPD with hazard ratios of 1.72, 1.67, and 2.29-3.10, respectively.

**Table 2:** Relative morbidity hazard estimates; overall, stratified by sex and the age group

			By sex				By age group			
				
Exposure		Overall (n = 2676)		Male (n = 871)		Female (n = 1806)		< 70 yo (n = 1323)		≥ 70 yo (n = 1354)
**Marital status after divorce/bereavement**										
remarried	0.51	(0.28-0.94)	0.67	(0.31-1.43)	0.36	(0.13-0.98)	0.36	(0.17-0.79)	1.26	(0.49-3.23)
not-remarried	1.00	(reference)	1.00	(reference)	1.00	(reference)	1.00	(reference)	1.00	(reference)
**Age at study entry**										
(continuous)	1.01	(0.99-1.02)	1.01	(0.99-1.03)	1.00	(0.99-1.02)	1.02	(0.99-1.06)	0.97	(0.93-1.01)
**Sex**										
male	1.03	(0.75-1.41)		N/A		N/A	0.81	(0.51-1.29)	1.24	(0.79-1.94)
female	1.00	(reference)					1.00	(reference)	1.00	(reference)
**Race/ethnicity**										
white/Caucasian	1.72	(1.10-2.68)	1.12	(0.58-2.16)	2.26	(1.21-4.24)	2.03	(1.10-3.75)	1.55	(0.80-2.99)
others	1.00	(reference)	1.00	(reference)	1.00	(reference)	1.00	(reference)	1.00	(reference)
**Education**										
high school or higher	1.00	(reference)	1.00	(reference)	1.00	(reference)	1.00	(reference)	1.00	(reference)
none/primary/secondary	1.67	(1.24-2.25)	1.57	(0.93-2.65)	1.72	(1.20-2.47)	1.93	(1.28-2.90)	1.36	(0.88-2.10)
**Number of cigarettes at study entry**										
none	1.00	(reference)	1.00	(reference)	1.00	(reference)	1.00	(reference)	1.00	(reference)
1-20/day	2.29	(1.57-3.34)	1.34	(0.66-2.75)	2.92	(1.87-4.57)	1.88	(1.17-3.01)	3.16	(1.73-5.77)
> 20/day	3.10	(1.63-5.89)	1.90	(0.67-5.42)	4.39	(1.96-9.84)	2.55	(1.13-5.76)	5.00	(1.76-14.23)

Data are hazards ratios (95% confidence interval) unless otherwise indicated.

The above-mentioned variables were all included in the model.

In stratified analysis, female subjects and under 70-year-old subjects manifested a significant difference in the incidence of COPD between those who remarried and those who remained single (hazard ratios: 0.36 and 0.36, respectively). In these subject groups, white/Caucasian race, lower education level and smoking more cigarettes had a significant increase of hazard ratios. Smoking cigarettes also showed a soaring incidence rate in subjects 70 or older.

## Discussion

The present study revealed that remarriage after bereavement or separation from one's spouse correlated with a reduced incidence rate of COPD. Our study model contained the number of cigarettes smoked for evaluating the impact of the remarriage effect in a more direct manner. And we included not only bereaved people but also divorced/separated people. Divorce/separation is difficult to confirm from death records or other national health statistics, and hence it has seldom been taken into account in previous studies. In addition, the marriage status variable was based upon *de facto *marital status, not legal status. Since marital status sometimes changes without changing legal status, our study model would be reflected in the actual realities of subject's marital situations[26].

In this study, white/Caucasian race, lower education level and smoking more cigarettes also significantly correlated with higher incidence of COPD. Previous studies revealed that smoking was a dominant risk factor for COPD and white/Caucasian race was prone to this disease because of a genetic susceptibility [27]. In addition, lower education level was also related to the high prevalence of COPD [28]. These results of earlier studies were consistent with the results of this study.

While the prevalence of COPD in this study was 14.6% (600/4100) just before the study entry, the estimated prevalence of COPD based on well-designed epidemiologic studies was in the range of 4% to 10% among people aged over 15 years [29]. In national statistics, the estimated prevalence of self-reported COPD was about 10% among people over 65 years [30]. Considering the majority of HRS participants are older adults, it would be safe to say the prevalence in this study was close to a reasonable range and indicated the external validity of this study to a certain extent. In addition, it could not be denied that misclassification might occur because the prevalence estimates of COPD based on self-reports. However, epidemiological surveillance of COPD was often conducted not only by physiologic pulmonary function tests (*e.g*. spirometry) but also by patient reports based on the diagnosis by attending physician, we, therefore, used self-reports in this study [29].

In regard to the association between marital status after bereavement and the incidence of diseases (*e.g*. COPD), two hypotheses are widely accepted in the bereavement studies: selection mechanisms hypothesis and social causation mechanisms hypothesis [19]. The selection mechanisms hypothesis proposes that the relatively good health status of married persons is the result of the selection of "healthy" persons into and "unhealthy" persons out of the married state, thus the relative amount of unhealthy person increases in the unmarried group. Applying this hypothesis to our study, bereaved or widowed people who were heavy smoker or have poor respiratory health tended not to remarry and, at the same time, are prone to COPD. On the other hand, the social causation mechanisms hypothesis advocates that marriage itself has a health promoting or a health protective effect while being unmarried would have adverse health effect. In this hypothesis, the effect of marital status on health is assumed to be intermediated by psychosocial factors (*e.g*. grief), the change of socio-economic status (*e.g*. income, burden of housework) and the alteration of health behaviors (*e.g*. smoking cessation). According to this hypothesis, remarried people after bereavement or widowhood tended not to develop COPD due to relief of stress, smoking cessation, and so on.

While two hypotheses are not mutually exclusive, it seems reasonable to suppose that social causation mechanisms hypothesis is more credible in this study. Because there was no significant difference of smoking rates between the unmarried and remarried group and the number of cigarette was included as a covariate in the model, we, therefore, considered that the influence of smoking which was a dominant confounding factor on the selection mechanisms hypothesis was not so great in this study. Although it is difficult to provide concrete descriptions concerning social causation mechanisms hypothesis on COPD, we assumed that changing health behavior after remarriage is the most probable cause. Remarriage after bereavement or widowhood may bring smoking rates lower because smokers who remarry after bereavement tend to quit smoking with consideration for or request from their new partner. This leads to tobacco cessation in later life which may slow the progression of airway damage and hamper a 'final push' to the onset of COPD. Whereas the development of COPD is the result of life-long progression of pulmonary damage, it was previously reported that stress itself was a risk factor of worsened pulmonary obstruction [22,31]. It is, therefore, appropriate to adopt the social causation mechanisms as an explanation for the results in this study.

This study has some limitations. The HRS has been conducted every 2 or 3 years and, therefore, we could not identify the subjects who developed COPD within 2 or 3 years since they divorced or were bereaved. Second, the majority of subjects in this study were retirement age and so the remarriage effect in middle age and youth remains unclear. Third, some confounding factors remain unadjusted. In this study, we adjusted for race/ethnicity and education background as confounding factors, but other potential confounding factors such as income, bronchial hyper-responsiveness and so on.

## Conclusion

The present study revealed that remarriage after bereavement or separation from a spouse was associated with a significantly reduced incidence rate of COPD. Remarriage might have a positive effect on the health status concerning COPD, it is, however, uncertain how remarriage affects the changes in the health status in individual diseases. To accommodate an aging society, further studies will be needed to figure out the potential relationship between remarriage behavior and the alteration of the health status.

## Competing interests

The authors declare that they have no competing interests.

## Authors' contributions

Study conception and design: TN, RT and KN. Statistical analyses: TN and AH. Drafting the manuscript: TN, TO and SH. All authors have read and approved the final manuscript.

## Pre-publication history

The pre-publication history for this paper can be accessed here:

http://www.biomedcentral.com/1471-2458/9/412/prepub
